# Photothermal Release by Melanin-like Nanoparticles: Biomedical Applications

**DOI:** 10.3390/jfb16070243

**Published:** 2025-07-02

**Authors:** Arianna Menichetti, Silvia Vicenzi, Agata Pane, Dario Mordini, Fabrizio Mancin, Marco Montalti

**Affiliations:** 1 Department of Chemistry “Giacomo Ciamician”, University of Bologna, Via Selmi 2, 40126 Bologna, Italy; arianna.menichetti2@unibo.it (A.M.); silvia.vicenzi4@unibo.it (S.V.); agata.pane2@unibo.it (A.P.); dario.mordini2@unibo.it (D.M.); 2 Tecnopolo di Rimini, Via Dario Campana 71, 47922 Rimini, Italy; 3 Department of Chemical Sciences, University of Bologna, Via Marzolo 1, 35131 Padova, Italy; fabrizio.mancin@unipd.it

**Keywords:** PTT, PDT, controlled release, drug delivery, nanomedicine, synergistic therapy, multimodal, theragnostic, phototherapy, antioxidants

## Abstract

Melanin-like nanoparticles (NPs) exhibit a remarkable ability to absorb light across a wide range of wavelengths, from the ultraviolet (UV) to the near-infrared (NIR) spectrum. This characteristic enables them to serve as effective photothermal agents (PTAs). Upon irradiation, especially within the NIR window, a region where biological tissues are highly transparent, these NPs efficiently convert light energy into heat. This phenomenon, known as the photothermal effect, leads to localized temperature increases. The resulting heat can be strategically employed to induce selective cell death in photothermal therapy (PTT) or to enhance the release of therapeutic agents directly from the NPs. The inherent versatility of melanin-like NPs, stemming from their synthesis methods and the presence of various functional groups, allows for straightforward loading with drugs or other bioactive molecules. Consequently, they are attractive tools for photothermally activated release. This review paper thoroughly examines and critically discusses the latest applications of melanin-like NPs in photothermally controlled release. We dedicate a specific section to general mechanisms and approaches, and this paper concludes with an analysis of critical challenges and prospective future developments.

## 1. Introduction

**Figure 1:**
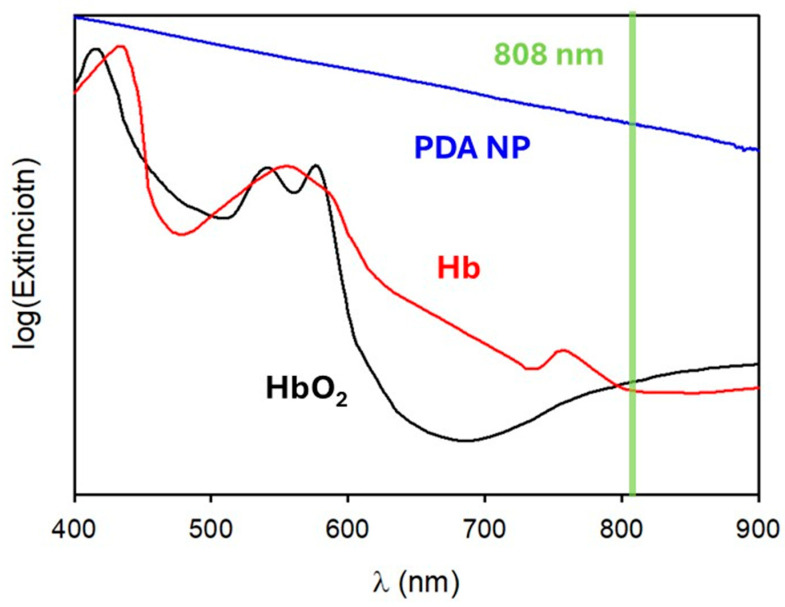
Extinction spectra (logarithmic scale) of PDA NP (blue line) and of the two main colored component of living tissues (hemoglobin, Hb, red line) and oxygenated Hb (HbO_2_, black line). In the NIR, and in particular using an 808 nm laser, PDA can be excited efficiently while the tissues show good transparency.

**Figure 2:**
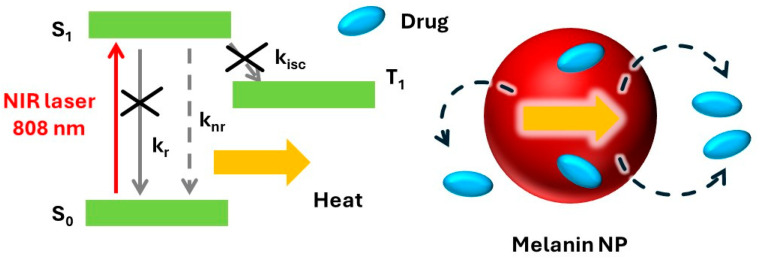
Scheme of the photo-release process. The simplified electronic state diagram (**left**) shows that when non-radiative deactivation is the dominant deactivation process (k_nr_>>k_r_ and k_nr_>>K_isc_, where k_r_, k_nr_ and K_isc_ are the kinetic constants for the radiative, non-radiative and inter-system-conversion processes, respectively), heat is released upon excitation. Temperature increases accelerate the release of drug molecules by the NP (**right**).

Chemically speaking, melanin is an umbrella term that encompasses a variety of colored biopolymers. In nature, it occurs in different forms and hues, and it has been found in several living beings (animals, plants, fungi and bacteria); however, the simplest and the most effective way to classify the different materials known as melanin is by the molecular precursors from which they originate [1]. We are talking about eumelanin, pheomelanin, neuromelanin, allomelanin and pyomelanin. These share similar physiological and biochemical functions such as the photoprotection and pigmentation of biological tissues. In both cases, these processes are driven by the interaction of the organism with sunlight [2,3]. The light-absorbing ability of melanin is well documented, and one of the main characteristics of this family of pigments is its broad absorption spectrum, which ranges from the UV to the near-infrared (NIR) region (see Figure 1) [4,5]. Although the term “melanin” was first introduced by Berzelius as early as 1840 [6], the actual composition and structure of this family of pigments is still only partially known. Indeed, the high degree of chemical disorder and the scarce processability of natural melanin have hampered its fine chemical analysis. The main challenge in the chemical characterization of melanin lies in its complexity, which arises from the co-presence of several different molecular units bound to each other both in a covalent and supramolecular way [7,8,9,10]. The limited knowledge of this material’s chemical heterogenicity restricts speculation regarding the origin of its unique optical properties. To elucidate this problem, ultrafast transient absorption (UFTA) spectroscopy was recently used to investigate the origin of the optical transitions responsible for the broad band absorption of melanin. In particular, it has been involved in distinguishing between transitions localized on specific chromophores and charge-transfer transitions involving the interaction between several chromophores [11,12,13,14,15,16]. Although different models have been proposed, the co-existence of localized and delocalized optical transitions is widely accepted, even if the relative contributions of the two kinds of processes are strongly dependent on the kind of sample analyzed. Within this variability, all the authors agreed in classifying the main deactivation path of the excited state as a fast (picosecond time range), non-radiative process. This behavior explains the good photoprotective activity of melanin since light energy is dissipated fast and safely as heat without a substantial formation of photoreactive species. This observation is in agreement with the scarce photoreactivity of melanin; in fact, its photochemical processes have been always characterized by a very low quantum yield [17]. As schematized in Figure 2, the fast thermal deactivation of the excited states, on the other hand, is known to be responsible for the photothermal effect, and hence heat release, upon light irradiation.

### 1.1. Natural and Biomimetic Melanin

Natural melanin results from enzymatic oxidation and the polymerization of tyrosine, which occurs in specialized cells called melanocytes and hence is transferred in the form of melanosomes to other cells, such as keratinocytes [8]. Melanin is also the main component of the black-colored nanoparticles (NPs) constituting the ink of several mollusks including cuttlefish (*Sepia Officinalis*). Mimicking the biological processes that lead to the formation of melanin, different kinds of melanin were prepared artificially, starting from different molecular precursors [6,18]. The most typical example of these processes is the synthesis of polydopamine NPs, which starts with an aqueous solution of dopamine, a base (ex. NaOH, NH_3_), and uses atmospheric oxygen as an oxidant agent at room temperature [18]. This process is extremely environmentally friendly and highly versatile, allowing a simple and direct functionalization of the produced NPs.

### 1.2. Photothermal Processes

The photothermal (PT) effect is the release of heat by a molecular or nanosized agent (the photothermal agent, PTA) under light irradiation. The main application of this effect in nanomedicine is the use of local heating to kill malignant cells such as cancer cells. The therapeutic process based on the PT effect is called PT therapy (PTT) [19,20]. PTT presents some advantages that are common to photodynamic therapy (PDT), where a molecular or nanosized agent, called photosensitizer (PS), is excited with light and reacts with oxygen to produce radicals via electron transfer (type I PDT) or gives a singlet oxygen (^1^O_2_) via excited state energy transfer [21]. These advantages comprise (i) the possibility of killing cells selectively by localizing light excitation using a collimated laser beam, for example, or (ii) by controlling the accumulation of the photoactive agent in specific cells, exploiting molecular recognition or simply the enhanced permeability and retention (EPR) effect [22,23,24]. Moreover, activation can be controlled in time by switching ON/OFF a continuous light source or by using a pulsed one. On the other hand, one of the main drawbacks of PDT is the poor concentration of oxygen typical of most cancer tissues. This situation, referred to as hypoxia, makes PDT poorly efficient [25]. Low concentrations of oxygen, on the other hand, are not problematic for PTT, which can, hence, represent a promising alternative to PDT. Both PTT and PDT present as a critical limitation the poor transparency of living tissues to light. For this reason, the light wavelength that has been selected to activate the system is crucial since the biological matrix is more transparent in the NIR region and much less in the UV-Vis region. To exploit the PT effect, the PA needs to present some additional features like the efficient non-radiative deactivation of the excited state and good photostability. For these reasons, plasmonic nanostructures, typically based on gold nanorods, are widely used for PTT [24,25,26]. Nevertheless, melanin nanostructures promise to be a valid alternative due to their superior biocompatibility when they are compared to metal nanomaterials [27].

### 1.3. Photoacoustic Imaging

A considerable advantage of the application of nanostructures to medicine is the possibility of developing multifunctional platforms that combine therapeutic action with diagnostics in a theranostic approach. Highly efficient therapeutic methods, like PTT, are also potentially hazardous and require continuous feedback for real-time control; hence, the combination of therapy with diagnostics is highly advantageous, especially because the same light stimulus can be used for both processes [28,29]. Photothermal heat release is associated with the generation of an acoustic wave that can be detected as a diagnostic signal. Moreover, by scanning the excitation in a controlled way, the origin of the acoustic wave can be localized to give an image of the target. This technique, known as photoacoustic imaging (PAI), is often used in combination with PTT for diagnostic purposes.

### 1.4. Photothermal Release

As discussed above, the main purpose of this review paper is not to focus on PTT but on the use of the PT effect to activate the release of therapeutic agents from melanin-like NPs used for PTA. This process is schematized in Figure 2. In particular, bioactive agents (e.g., drug molecules) can be loaded onto the NPs. Their release to target tissues is accelerated upon heating because of the photothermal effect, leading to a synergistic therapeutic effect.

In the next paragraphs, the main nanomedicine applications of melanin-like NPs photothermal properties will be highlighted considering the most relevant examples published in the last five years. Among them, we will show that cancer treatment and tumor ablation have been highly investigated in the scientific community. Moreover, the antibacterial, wound healing and antioxidant capabilities, as well as other bio-medicine applications, of the NPs will be described.

## 2. Cancer Treatment

To address the limitations of conventional cancer therapies, current research is focused on the development of new stimulus-responsive drug delivery systems. These innovative systems are engineered from various nanostructured materials that are able to respond to specific endogenous or exogenous stimuli, such as the pH of the tumor environment, exposure to near-infrared (NIR) irradiation or other physical alterations within the tumor surroundings. One of the primary objectives of researchers has been to enhance therapeutic efficacy through the integration of synergistic treatment modalities, including chemotherapy (CT), PTT, PDT, chemodynamic therapy (CDT), gas therapy (GT) and others. These therapeutic modalities are frequently coupled with various imaging techniques, including magnetic resonance imaging (MRI) and fluorescence imaging (FLI) [30], to facilitate the real-time monitoring of experimental efficacy. Various cancer types have been investigated and treated with different strategies that exploit the photothermal properties of PDA NPs.

Common anticancer drugs include metformin (Met) and doxorubicin (DOX). However, when orally administered, they show poor bioavailability and potential toxicity [31]. Thus, they have been loaded into different nano-systems to improve their pharmacokinetic profiles and enable their controlled release at the tumor site.

**Figure 3:**
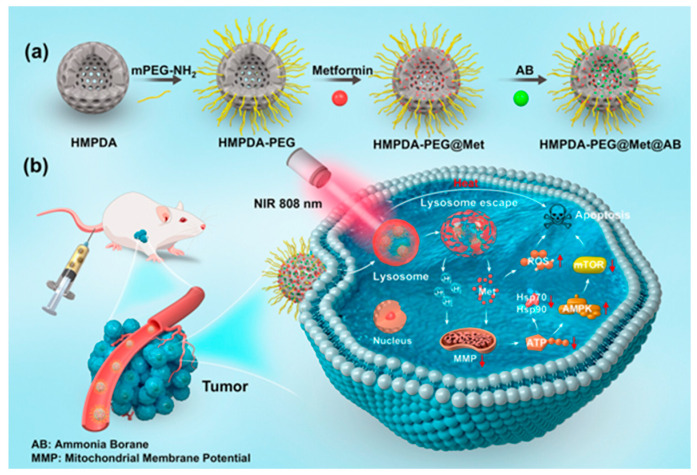
(**a**) Scheme flowchart depicting the synthesis of HMPDA-PEG@Met@AB; (**b**) mechanisms of action of metformin and Ammonia Borane co-delivered for melanoma therapy. Reprinted with permission from Ref. [32]. Copyright 2025 American Chemical Society.

A recent study by Lin et al. demonstrated that hollow mesoporous polydopamine nanocomposites co-loaded with Met and Ammonia Borane (HMPDA-PEG@Met@AB released Met and H_2_ when internalized in the acidic tumor environment, thereby inducing apoptosis in tumor cells in the case of melanoma. H_2_, derived from the Ammonia Borane, was essential for enhancing the nanocomposites’ escape from lysosomes and for inhibiting heat shock protein expression in mouse melanoma B16−F10 cells (see Figure 3). The release is synergistically triggered by the acidic pH, high concentration of reactive oxygen species (ROS) and exposure to 808 nm NIR irradiation, which is amplified by the photothermal effects of HMPDA. The in vivo and in vitro tests showed significant antitumor effects, good biocompatibility and a low toxicity of the developed nanoplatform [32].

Zhang et al. loaded DOX into polydopamine/gold (Au) hollow nanospheres (HPDA/Au@DOX) for the treatment of non-small cell lung cancer [33]. Similarly, DOX was loaded into a nanofibrous scaffold composed of poly(ε-caprolactone) and hydroxyapatite coated with polydopamine (PCLDH@PDA) to treat osteosarcoma and enable bone regeneration after osteosarcoma surgery by Rezk and colleagues [34]. Furthermore, DOX was included in a novel oral nano-drug delivery system (MPDA-FA-DOX/ES100) consisting of mesoporous polydopamine NPs modified with folic acid (FA) for targeting colon cancer cells overexpressing folate receptors by Gu et al. [35]. These studies highlighted the effectiveness of the combined therapy approach involving CT with DOX and PTT using PDA NPs. Indeed, these in vitro studies demonstrated that the CT/PTT combination resulted in increased cytotoxicity and stronger antitumor effects on cancer cells compared to CT alone in A549 lung cancer cells and CT26 colon cancer cells. In the case of the nanofibrous scaffold for the treatment of osteosarcoma, in vitro tests showed the enhanced adhesion, proliferation and osteogenic differentiation of human mesenchymal stem cells, and also promoted new bone biomineralization leading to natural apatite formation. In vivo tests of the first and third studies showed improved antitumor efficacy and the good biocompatibility of the developed systems.

A series of other scaffolds involving DOX and polydopamine have been reported in the most recent research articles. For example, Xu et al. reported the development of a 3D-printed heterogeneous hybrid hydrogel scaffold designed to both prevent melanoma recurrence and promote wound healing after melanoma surgery. The scaffold was composed of sodium alginate (SA), gellan gum (GG) and PDA NPs (SA-GG@PDA) used as a coating for a DOX-loaded thermosensitive gelatin hydrogel. Another study by Liu et al. presented a Gel/polycaprolactone (PCL)/PDA core/shell fiber scaffold fabricated using 3D printing for NIR-triggered on-demand drug release. The core was made of alginate/gelatin hydrogel loaded with DOX, and it was coated with a PCL layer to reduce uncontrolled drug diffusion. Subsequently, a PDA coating was added to provide photothermal effects, allowing NIR laser irradiation to induce a sol–gel transition in the core and subsequent drug release. Both studies demonstrated accelerated DOX delivery and effective CT/PTT effects under NIR irradiation, leading to the significant suppression of tumor cell proliferation. The SA-GG@PDA+DOX hydrogel scaffold promoted the proliferation and migration of human umbilical vein endothelial cells (HUVECs), enhancing wound healing in a mouse model. In vivo experiments confirmed the positive results of the combined treatment, with the inhibition of tumor recurrence and efficient wound closure [36,37]. As evident from previous studies, facilitating wound healing is a major priority when treating cancer patients undergoing surgery. Lei and colleagues presented the fabrication of hyaluronic acid (HA)-based microneedle (MN) patches functionalized with biomineralized melanin nanoparticles encapsulated in an amorphous silica (SiO_2_) shell (CINP@SiO2-HA MNs), designed for achieving both the PTT of skin tumors and subsequent wound healing. The melanin nanoparticles, naturally derived from cuttlefish ink (CINP), exhibited antioxidant and photothermal properties and were able to release bioactive SiO_4_^4−^ ions, known to stimulate skin tissue regeneration. Moreover, the MN patches enabled efficient skin penetration, allowing for the eradication of residual tumor cells via PTT and the inhibition of bacterial infection. Additionally, the combined antioxidant effects of melanin and the release of SiO_4_^4−^ helped modulate inflammation and stimulated angiogenic gene expression, supporting tissue repair and regeneration [38]. Tumor targeting can be enhanced by modifying the surface of the nanoplatform, as demonstrated by Lv and colleagues. They developed a “carrier–drug” hybrid assembly using PDA and DOX for enhanced cancer therapy, modifying the surface with iRGD-apoA-I, a tumor-penetrating peptide that binds to receptors overexpressed on tumor blood vessels and helps the substances move deeper into the tumor tissue. The resulting nanoparticles exhibited triple-responsive drug release triggered by acidic pH, ROS and NIR light. In vitro and in vivo studies demonstrated efficient tumor penetration and accumulation, leading to significant tumor growth and metastasis suppression via synergistic CT/PTT in a 4T1 orthotopic tumor-bearing mouse model [39].

Beyond Met and DOX, other therapeutic drugs, derived from natural sources, have been tested for the treatment of cancer. Annonaceous acetogenins (ACGs), a group of naturally occurring compounds with cytotoxic effects, have been integrated with PEGylated PDA NPs for breast cancer treatment. While ACGs possess significant antitumor activity, they also exhibit considerable systemic toxicity, which this delivery system aimed to address. The combination of ACG NPs and PDA-PEG NPs with NIR irradiation resulted in a significant survival rate reduction in 4T1 cell viability in vitro. In vivo studies showed that the intra-tumoral injection of this combined system achieved a significantly higher tumor inhibition rate compared to ACG NPs alone, effectively reducing the systemic toxicity associated with ACGs through localized delivery [40]. Considering another active biomolecule, baicalein (BA) is a natural flavonoid compound widely studied for its anti-inflammatory, antioxidant and anticancer properties [41]. It was loaded into pH/photothermal-responsive ZIF-8 nanocarriers (BZPP (BA@ZIF-8-PDA-PEG)) for synergistic cancer therapy by Gao et al. This BZPP exhibited pH-triggered drug release in the acidic environment of tumor cells. In vitro studies on A549 cells revealed efficient cellular uptake and the enhanced synergistic antitumor effects of combined CT/PTT compared to monotherapy [42]. A natural compound known for its anti-inflammatory, antioxidant, anticancer and antibacterial properties is curcumin. This was the key chemical compound of a drug delivery system developed by Zhao et al. Their study outlined the fabrication of a system able to release curcumin, which was loaded into mesoporous polydopamine nanoparticles. This system was subsequently encapsulated within a hydrogel formed through the cross-linking of oxidized konjac glucomannan (oxKGM) and carboxymethyl chitosan (CMCS). The release kinetics of curcumin were accelerated upon NIR irradiation and the acidic pH of the tumor environment due to the pH-sensitivity of the hydrogel. The developed hydrogel showed good biocompatibility and significant toxicity against murine 4T1 breast tumor cells [43]. In a separate publication, Ma and colleagues explored an alternative trigger modality for breast cancer treatment. They created a NIR-triggered phase-transition material-based nanoplatform modified with polydopamine and loaded with DOX (PDA-M@PCM(DOX)). The increase in temperature triggered by NIR irradiation led to a phase change in the material, enhancing the controlled drug release at the tumor site, obtaining a significant in vitro and in vivo cell killing activity of 4T1 breast cancer cells [44].

Beyond the combination of CT with PTT to treat cancer, scientists are focusing on exploring novel strategies for cancer cell death by coupling this technique with others such as (i) immunotherapy, a type of cancer treatment that uses the body’s own immune system to recognize, attack and destroy cancer cells; immunogenicity, which relies on the ability of a substance to cause an immune response in the body; (ii) PDT, which uses light-sensitive compounds, light and oxygen to kill cancer cells; (iii) nitric oxide GT, which exploits the vasodilatation, immune regulation, neurotransmission and cell signaling power of nitric oxide, leading to cell apoptosis; and (iv) CDT, which relies on the production of toxic free radicals at the tumor site able to kill cancer cells by damaging their DNA, proteins and membranes. Immunotherapy was investigated by Chen and colleagues for the treatment of anaplastic thyroid carcinoma. They coupled the chemotherapeutic effect of DOX with the immunotherapeutic potential of double-stranded CpG oligodeoxynucleotides (dsCpG ODNs) for the development of a novel multifunctional nanoplatform, loading the drugs into polydopamine nanoparticles (PDA-dsCpG-DOX NPs). Upon systemic administration, the dsCpG ODNs mimic bacterial DNA, triggering an innate immune response that can be enhanced upon irradiation with an 808 nm NIR laser, which also triggers the photothermal release of DOX for chemotherapy. In vivo experiments using a mouse model bearing subcutaneous xenograft ATC tumors demonstrated that treatment with PDA-dsCpG-DOX NPs and the NIR laser resulted in nearly complete tumor growth suppression and stimulated a systemic antitumor immune response, exhibiting good biocompatibility [45].

Notably, immunogenicity can be correlated to ferroptosis, a form of regulated cell death characterized by iron-dependent lipid peroxidation, initiated by the accumulation of ROS, which leads to lipid oxidation, causing cell damage and death [46]. However, ferroptosis can be considerably limited when lipid peroxidation (LPO) is not high enough. To increase LPO, different techniques have been investigated. Clustered Regularly Interspaced Short Palindromic Repeats (CRISPR), a powerful gene editing technology that allows the precise modification of DNA within living organisms, has been explored in conjunction with nanoparticles and hydrogels to deliver gene editing tools directly to tumors, enabling targeted gene regulation and minimizing off-target effects. CRISPR has been evaluated as a tool for enhancing ferroptosis for the treatment of cancer by Pan and colleagues, who introduced a novel engineered bacteria-derived nanocluster using T7 polypeptide-engineered bacterial outer membrane vesicles coupled with the CRISPR system and with melanin nanoparticles (TO@Mp) to obtain LPO amplification and consequent ferroptosis. CRISPR was employed as a gene editing tool to inhibit glutathione peroxidase 4 (GPX4), a key enzyme that inhibits LPO, thus amplifying its accumulation. Simultaneously, the photothermal treatment generated reactive oxygen species (ROS), further cooperating with GPX4 knockdown to reinforce ferroptosis upon NIR laser irradiation. In vitro and in vivo experiments demonstrated that the TO@Mp nanoparticles augmented immunogenic cell death (ICD) via this synergistic lipid peroxidation-induced ferroptosis [47].

**Figure 4:**
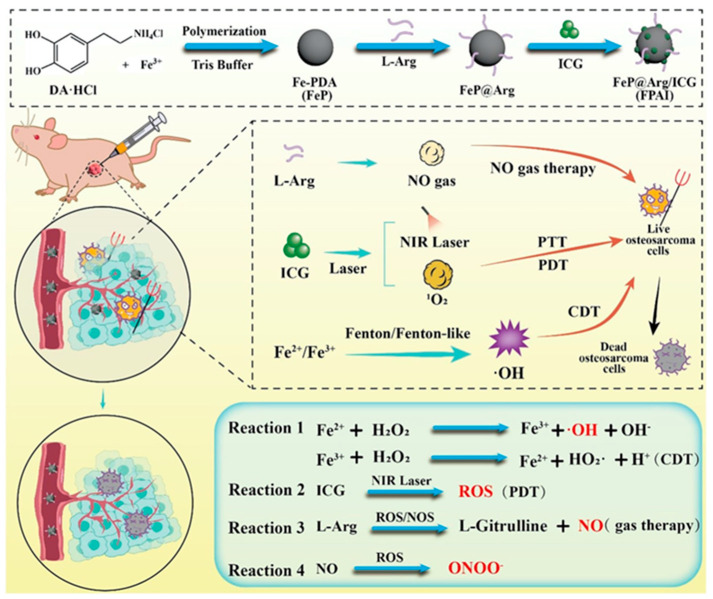
The synthetic process of FPAI NPs and the schematic diagram of NIR-induced FPAI NPs to achieve PTT/PDT/CDT/NO gas combination therapy for osteosarcoma in vivo. Reprinted with permission from Ref. [48]. Copyright 2025 Elsevier.

Regarding other strategies, Yuan and colleagues integrated many of the mentioned techniques for osteosarcoma treatment. They developed polydopamine-based multifunctional nanoparticles (FPAI NPs) consisting of iron and polydopamine, coated with the nitric oxide initiator L-Arginine (L-Arg) and the photosensitizer indocyanine green (ICG). These components were designed to work synergistically to combine the effects of PTT, PDT, CDT and GT. The presence of hydrogen peroxide (H_2_O_2_) and the acidic pH within the tumor microenvironment facilitated Fenton/Fenton-like reactions upon the release of iron ions, thus enhancing CDT. NIR laser irradiation activated/enhanced all four therapies, ICG mediated PTT/PDT and L-Arg generated nitric oxide for GT. This synergistic “PTT/PDT/CDT/GT” strategy demonstrated significant tumor growth suppression in a 143B mouse osteosarcoma model (see Figure 4) [48].

PDT coupled with CT and PTT using PDA NPs was further assessed by Pal and co-workers, who developed a novel nanotherapeutic agent (FA@CuO@Ce6-PDA/PTX) employing chlorin e6 (Ce6) as a photosensitizer, loaded with Paclitaxel (PTX) for CT and conjugated with folic acid (FA) for targeted delivery to cancer cells. In vitro studies on 4T1 cells demonstrated significant synergistic antitumor effects with CT/PTT/PDT upon NIR laser irradiation, showing increased ROS production and cell death [49]. Similarly, Gong et al. developed a temperature-responsive composite hydrogel for controlled drug release and synergistic PTT and PDT. The hydrogel (CSZP) was composed of two-dimensional polydopamine (2D PDA), chitosan (CTS), zinc phthalocyanine (ZnPc) as a photosensitizer and sodium percarbonate (SPC) as an oxygen donor. Their research revealed that upon near-infrared (NIR) laser irradiation, the 2D PDA efficiently converted light into heat, causing the hydrogel to disintegrate and release ZnPc and oxygen. The released oxygen mitigated tumor hypoxia (absence of oxygen), enhancing the efficacy of PDT mediated by ZnPc, which generated ROS upon irradiation with 665 nm light. In vitro and in vivo experiments on 4T1 breast cancer cells and tumor-bearing mice demonstrated significant tumor growth inhibition with the CSZP hydrogel under combined PTT and PDT, in addition to good biocompatibility and the controlled release of therapeutics for targeted disease treatment [50]. A different strategy to harness the potential of PDA NPs was employed by Getachew et al., using them for coating defect-passivated CsPbBr_3_ quantum dots (QDs), specifically lead halide perovskite nanocrystals, with excellent optical and electronic properties, using diammonium sulfide [(NH_4_)_2_S] as a passivating agent able to reduce the defects and loading them into porous manganese functionalized with FA to obtain multifunctional nanospheres (NCPB@mPDA/FA NSs). Under acidic conditions, Mn^2+^ ions generated cytotoxic hydroxyl radicals (•OH) for CDT, while S^2−^ ions produced H_2_S gas for GT. Upon 808 nm laser irradiation, the nanospheres demonstrated excellent PTT and triggered the release of Mn^2+^ and S^2−^ ions for enhanced CDT and GT [51]. Many other recent studies have focused on coupling different techniques and integrating different materials into novel nanoplatforms to exploit the properties of PDA NPs. For instance, Ma et al. developed a multifunctional nano-system using dendritic mesoporous silica nanoparticles (DMSNs) as carriers for CuO_2_ nanodots (for CDT) and probes or ROS monitoring for treating breast cancer [52]. Chen and colleagues developed a multimodal synergistic nanoplatform with dual NIR and pH responsiveness using PCN-600 (Porous Coordination Network, composed of Fe ions and a TCPP ligand) as a carrier for DOX and PDA, able to generate singlet oxygen at the tumor site, enhancing PDT under 633 nm laser irradiation [53]. Shi et al. developed a nanostructure consisting of silica nanoparticles loaded with DOX and coated with PDA, MnO_2_ and hyaluronic acid able to release Mn^2+^ ions for CDT enhancement [54]. These studies collectively highlight the positive outcomes of combined CDT/PTT/PDT strategies regarding controlled drug release upon NIR irradiation and in response to the acidic tumor environment, resulting in significant antitumor efficacy.

Cancer treatment not only involves therapeutics but also plays a major role in diagnostics; thus, it is possible to talk about theranostics, a medical field that couples the two. Diagnostics is often exploited through cancer imaging, a technique involving the visualization and monitoring of tumors, allowing the detection of tumors and the assessment of their progression during the treatments. Ma et al. introduced manganese sulfide nanoclusters coated with polydopamine and included magnetic resonance imaging (MRI), an imaging technique employing a strong magnetic field and radio waves to generate detailed images of the tumor, to integrate both diagnostics and therapeutics. The polyhydroxy structure of PDA coatings enhanced the interaction of water molecules with MnS nanoclusters through hydrogen bonding, leading to improved MRI resolution. Moreover, at a pH of 5.5, typical of the tumor environment, the spin-lattice relaxation rate, important to obtain more clear and detailed images, increased. Furthermore, the nanoclusters showed excellent biocompatibility and good photothermal efficiency. The MnS core decomposed in the acidic pH of the tumor environment and released H_2_S, able to inhibit mitochondrial respiration and ATP production in tumor cells, decreasing the resistance of tumor cells to photothermal stimulation and enhancing PTT. Lastly, the Mn^2+^ ions also exhibited efficient peroxidase and glutathione oxidase-like activities, leading to the ferroptosis and apoptosis of cancer cells [55].

**Figure 5:**
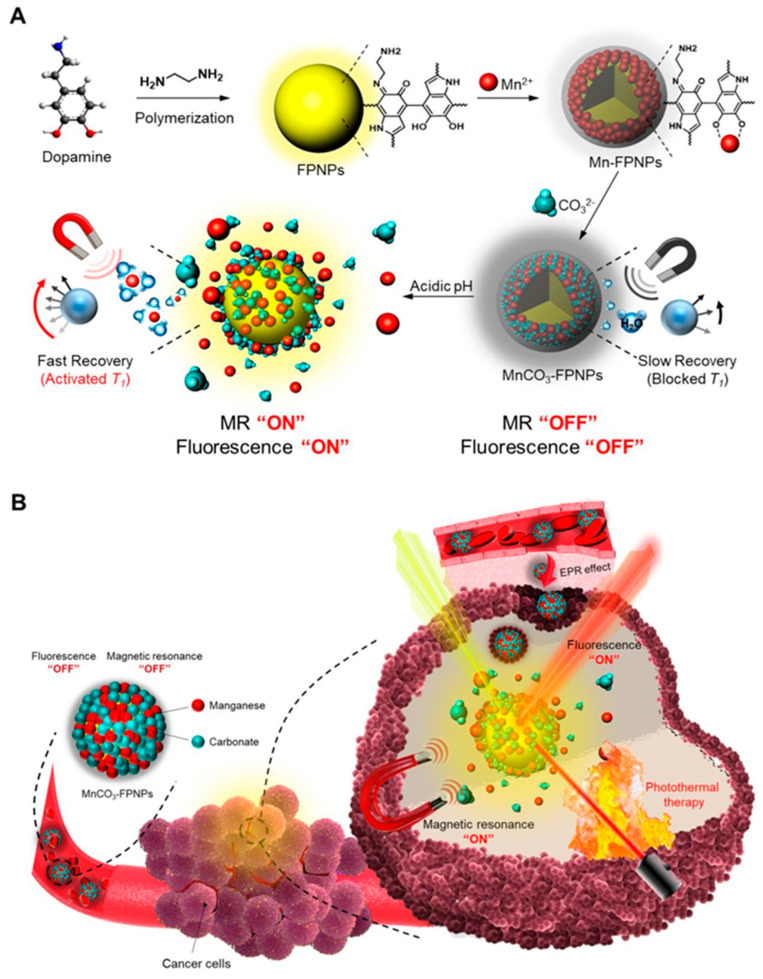
(**A**) Overall synthetic methods and working principle of MnCO_3_-FPNPs for activatable FL/MR dual-modality imaging. (**B**) Postulated mechanism of MnCO_3_-FPNPs for FL/MR imaging-guided PTT of tumors after tumoral accumulation by enhanced permeability and retention (EPR) effect. Reprinted from Ref. [57].

MRI was also employed by Jin and colleagues who developed a multifunctional nanocomposite (PION@PDA-PEG) that integrates MRI, PTT and CT for cancer treatment. Porous iron oxide nanoparticles (PIONs) were synthesized, coated with a PDA shell and loaded with DOX, and the nanocomposite surface was further modified with PEG to enhance blood circulation time. The resulting PION@PDA-PEG exhibited excellent biocompatibility and demonstrated promising activity as an MR imaging contrast agent. In vitro experiments demonstrated a synergistic anticancer effect by combining PTT and chemotherapy, significantly reducing cancer cell viability upon NIR irradiation. The nanocomposite also showed pH-responsive and NIR-triggered drug release behavior, potentially enhancing drug delivery to the tumor site [56]. MRI was again involved, together with fluorescence imaging (FLI), in a study by Lee et al., in which they developed MnCO_3_-mineralized polydopamine nanoparticles (MnCO_3_-FPNPs) as activable theranostic agents for dual-modal imaging-guided PTT. These nanoparticles exhibited a unique dual-modal FL (fluorescence)/MR (magnetic resonance) imaging capability, becoming active in the acidic tumor environment. At physiological pH (7.4), FL was efficiently quenched, and MR activity was silenced, whereas at acidic pH (5.4), a strong FL recovery and an enhancement in T1-weighted MR contrast occurred due to the release of Mn^2+^ ions. In vivo experiments on 4T1 tumor-bearing murine models showed tumor-specific accumulation thanks to the “OFF-ON” modes of FL/MR imaging, leading to significant tumor growth suppression after imaging-guided PTT. Surface mineralization with MnCO_3_ enhanced nanoparticle stability in serum conditions and improved the tumor-specific activation of FL (see Figure 5) [57].

As a fundamental advantage, FLI is a non-invasive optical imaging technique. Moreover, dyes emitting in the NIR region are preferred for bio-applications since biological tissues are more transparent in this spectral window. Gao et al. used Cy5.5, a near-infrared fluorescent dye, for FLI. They fabricated poly (acrylic acid)-mesoporous zinc phosphate/polydopamine (PAA-mZnP/PDA) Janus nanoparticles (JNPs) as biosafe PTT agents and pH/NIR-responsive drug carriers. FLI was primarily employed in their study to evaluate the biodistribution of the nanoparticles (JNPs) in vivo, specifically to determine their accumulation sites following administration in tumor-bearing mice. The results revealed that PAA-mZnP/PDA-PEG JNPs exhibited prolonged tumor accumulation, suggesting their potential for sustained therapeutic effects and passive tumor-targeting through the enhanced permeability and retention (EPR) effect [58]. A multifunctional theranostic nanoplatform (MGPO NPs) using the PAI technique was developed by Lian and colleagues. The nanocluster included glucose oxidase (GOx), oxygenated perfluoropentane (PFP) and mesoporous PDA NPs. PAI is a hybrid imaging modality that combines optical and ultrasound techniques and relies on the use of photoacoustic agents (PFP in this case) that can absorb pulsed laser light and convert it into ultrasonic waves via thermoelastic expansion, which can then be detected to form high-resolution images of tissues. NIR irradiation, absorbed by PDA NPs, is essential for increasing the temperature and enabling oxygen release from PFP to enhance consequent imaging. The supplied oxygen alleviates hypoxia, enhancing the catalytic activity of GOx for glucose deprivation therapy. MGPO NPs enabled ultrasound/PA imaging-guided synergistic therapy to improve tumor treatment efficacy. In vitro and in vivo studies demonstrated the ability of MGPO NPs to inhibit tumor growth and overcome tumor environmental challenges [59]. PAI was also assessed by Yu et al., who developed a pH-responsive UiO-66 framework loaded with DOX and coated with PDA for synergistic CT/PTT/PDT, which exhibited a triple-responsive drug release triggered by pH, glutathione and an NIR laser. A high photothermal conversion efficiency allowed photoacoustic imaging-guided diagnosis and treatment, along with significant tumor growth inhibition compared to monotherapies [60]. Ultrasound (US) imaging is a non-invasive diagnostic technique that uses high-frequency sound waves to produce real-time images of structures inside the body. Chen et al. presented a multifunctional nanoplatform (CQ@ICG@PFH@PDA-PEG5k) for US imaging and the enhanced PTT of triple-negative breast cancer (TNBC) by inhibiting autophagy. The nanoplatform was based on perfluorohexane (PFH) nanodroplets loaded with the autophagy inhibitor chloroquine (CQ) and the photosensitizer indocyanine green (ICG). Upon NIR laser irradiation, PFH underwent a liquid–gas phase transition, enabling US imaging, while ICG and PDA facilitated PTT. The released CQ inhibited autophagy, synergistically enhancing the tumor-killing effect of PTT [61].

Overall, these studies highlight the versatility of PDA NPs as a platform for the controlled photothermal release of anticancer drugs. Moreover, the ability to combine PTT with other therapeutic modalities such as chemotherapy, photodynamic therapy, chemodynamic therapy and immunotherapy represents a promising approach to overcome the limitations of conventional therapies and improve treatment efficacy across various cancer types. All the systems discussed in the above section are summarized in Table 1.

## 3. Antibacterial Applications

The development of new and pioneering antibacterial materials is acquiring a significant role due to the escalation of global concerns about bacterial infections and antibiotic resistance. Indeed, consolidated antibacterial treatments are often associated with limitations such as inadequate efficacy, the reinforcement of bacterial resistance and systemic toxicity. Recently, a novel approach combining controlled drug release with PTT emerged as a promising path for overcoming these limitations. In this section, we discuss the central role of PDA in antimicrobial therapies, where it works both as a photothermal conversion agent and as a controlled drug release platform. All these studies share common goals, namely (i) developing more effective therapies, (ii) reducing bacterial resistance and (iii) improving infected wound healing.

In many cases, PDA is combined with silver nanoparticles (AgNPs) to harness synergistic antibacterial and photothermal properties. AgNPs, in fact, exhibit antibacterial activity thanks to the release of silver ions and the production of ROS. However, high concentrations of AgNPs in living tissues can be potentially cytotoxic. The conjugation of a second material to AgNPs may augment their antibacterial efficacy, thus enabling the administration of a reduced dosage and, consequently, mitigating cytotoxicity. In 2021, Ma et al. developed an injectable adhesive antibacterial hydrogel where polydopamine-decorated silver nanoparticles (PDA@AgNPs) were introduced into a hydrogel network consisting of oxidized alginate (ADA) and catechol-modified gelatin (Gel-Cat), conferring excellent adhesiveness (GFA/PDA@AgNPs). This hydrogel’s polymer backbone, crosslinking in situ through double dynamic bonds, achieved injectability, short gelation times and improved mechanical strength. The PDA@AgNPs within this system functioned dually as a bactericidal agent, releasing ions to eliminate bacteria, and as a photothermal agent, converting 808 nm near-infrared light into heat for sterilization [62]. Wang et al. obtained similar results by developing a pH-responsive cationic guar gum-based multifunctional hydrogel that incorporated polydopamine-coated silver nano-enzymes (PDA/PM-AgNPs). These PDA/PM-AgNPs exhibited enhanced peroxidase-like activity at a mildly acidic pH and a high photothermal conversion efficiency, primarily attributed to the PDA shell. These PDA-coated nanoparticles also demonstrated superior radical scavenging activity, crucial for promoting wound healing. The hydrogels exhibited excellent in vitro antibacterial activity against *MRSA* and *E. coli*, which was facilitated by the mechanical disruption of biofilms, direct damages to the cellular structures and the induction of oxidative stress. In addition, in vivo evaluations highlighted the hydrogel’s efficacy, promoting angiogenesis and collagen synthesis, both crucial for wound healing [63].

**Figure 6:**
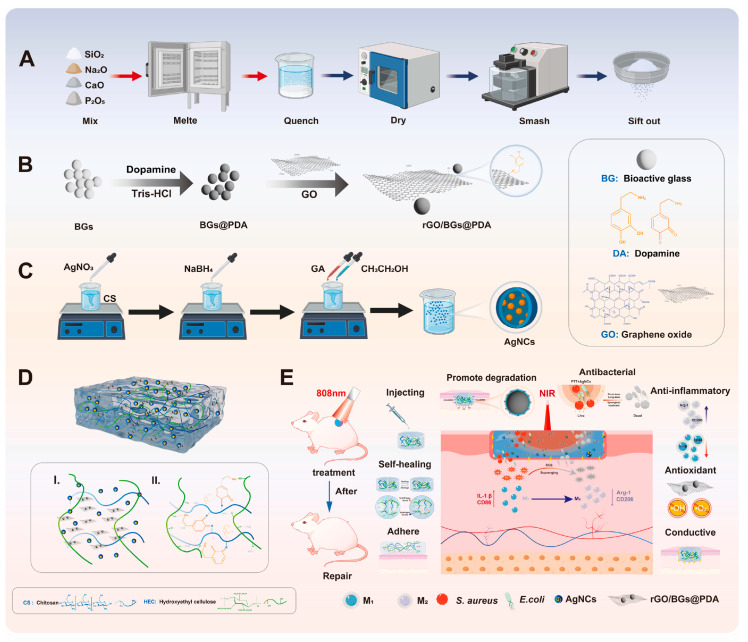
Schematic illustration of formation and application of rGO/BGs@PDA-loaded CS-HEC/AgNCs hydrogels for wound healing. (**A**) Synthesis of BGs. (**B**) Synthesis illustration of BGs@PDA and loaded on GO (rGO/BGs@PDA). (**C**) Synthesis of chitosan stabilized AgNCs. (**D**) Characteristics of hydrogels as well as local enlargements of composition (I) and the detailed crosslinking between the individual components (II). (**E**) Diagram of the mechanism of the hydrogel in wound healing. Reprinted from Ref. [64].

In combination with AgNPs, other materials have been explored. Song et al. introduced a photothermally enhanced hydrogel based on chitosan (CS) and hydroxyethyl cellulose (HEC) loaded with polydopamine-coated bioactive glass (BGs@PDA) on reduced graphene oxide (rGO) and silver nanoclusters (AgNCs) for synergistic antimicrobial treatment and wound healing promotion. This hydrogel leveraged PTT mediated by rGO/BGs@PDA, which exhibits high photothermal conversion efficiency, in combination with the antimicrobial activity of AgNCs for rapid bacterial eradication and controlled release. PDA not only acted as a photothermal agent and enhanced the hydrogel’s adhesion and antioxidant properties but also facilitated the degradation of bioactive glass, resulting in enhanced wound healing through the ion-stimulated release of biofactors. In vitro, this hydrogel demonstrated excellent antibacterial activity and biocompatibility, while in vivo studies confirmed accelerated wound healing and potent synergistic antibacterial effects (see Figure 6) [64].

Expanding on this concept, researchers have explored the integration of metal–organic frameworks (MOFs) alongside PDA and AgNPs. MOFs are ordered porous materials with a high surface area and tunable properties, making them promising platforms for drug delivery and superior therapeutic agents. He et al. and Huang et al. developed antibacterial nanoplatforms based on MOFs, although they relied on distinct synthetic strategies and structural outcomes. He et al. employed *γ*-cyclodextrin-based MOFs (CD-MOF) as a template for in situ AgNPs synthesis, followed by PDA encapsulation to enhance water stability and impart photothermal capabilities. In contrast, Huang et al. utilized a one-pot synthesis to create coral-like MOF-PDA, embedding AgNPs within the hollow structure via their PDA-reducing ability. The antibacterial performance of both systems was enhanced under NIR laser irradiation, a result attributed to the photothermal conversion of PDA enhanced by the plasmon resonance of the AgNPs. However, He et al. focused on controlled Ag^+^ release through the MOF pores as an antibacterial chemical action, while Huang et al. emphasized the synergistic antibacterial effects of multiple metal ions within the ZIFL structure. Both nanoplatforms demonstrated significant bacterial inactivation, highlighting the efficacy of combining MOFs, PDA and AgNPs for advanced antibacterial applications [65,66].

Novel delivery mechanisms are also being investigated. Li et al. combined the synergistic effect of Ag^+^ ion release and PTT with nanomotor movement. The authors were able to successfully synthesize silver-loaded asymmetrical, jellyfish-like mesoporous polydopamine nanoparticles (PDA-NH2@Ag nanomotor). The nanomotor under NIR laser irradiation exhibited negative phototaxis motion, inducing autonomous motion and thereby enhancing contact with bacteria. At a high concentration, the system demonstrated an accelerated bacteria-killing rate against both Gram-negative and Gram-positive bacteria [67].

Furthermore, researchers are refining drug delivery systems by integrating therapeutic agents. In 2025, Yang et al. developed a dual-antibiotic hydrogel delivery system consisting of PDA-coated silver nanoparticles and levofloxacin. Levofloxacin was attached to the surface of the PDA-coated AgNPs, which were subsequently incorporated into carboxymethyl chitosan (CMCS) hydrogels. The PDA-shell functioned as a photothermal switch, enabling the responsive release of antibacterial drugs upon 808 nm NIR irradiation. This combination of antibiotic release with the photothermal effect of PDA demonstrated the effective elimination of a drug-resistant bacterium (*P. aeruginosa*), increased the permeability of pathogenic bacterial cell membranes and inhibited the formation of bacteria biofilms. Both in vitro and in vivo tests confirmed that the PDA-Ag@Levo/CMCS system combined with NIR irradiation led to excellent bactericidal performance and could reduce the inflammatory response; nevertheless, it increased collagen deposition and promoted angiogenesis, resulting in rapid wound healing [68].

Beyond antibacterial activity, researchers are exploring the application of PDA-based hydrogels for wound pain mitigation. Wu et al. extended the application of PDA-based hydrogels to include prolonged local analgesia. They developed an adhesive, photothermal hybrid hydrogel designed for both antibacterial activity and on-demand lidocaine delivery. This hydrogel demonstrated enhanced mechanical properties, reversibility, adhesion and self-healing. Notably, it exhibited superior photothermal conversion efficiency compared to pure PDA, facilitating effective drug release and pain relief [69]. In the same year, Rybak et al. dealt with pain relief by including an anti-inflammatory drug, ketoprofen, in the hydrogel. The researchers developed a stimulus-responsive, injectable and in situ-forming hydrogel with antibacterial, self-healing and drug-delivery properties. The hydrogel was composed of Pluronic F127 (PF127) and sodium alginate (SA), and it exhibited temperature-sensitive behavior. Polymeric short filaments (SFs) carrying ketoprofen, an anti-inflammatory, and stimulus-responsive PDA particles were integrated into the hydrogel. PDA particles facilitated the efficient conversion of NIR light into localized heat, enabling on-demand drug release and the effective inactivation of both Gram-positive and Gram-negative bacteria. The resulting material reached temperatures of 60 °C and 66 °C, achieving an almost complete eradication of *E. coli* and *S. aureus*, respectively, without the inclusion of any other antibacterial drugs or silver nanoparticles [70].

In addition to AgNPs, other NPs have been explored. Yan et al. wrapped a copper sulfide core in mesoporous PDA and subsequently modified it with cationic polyethyleneimine (MPDA@Cu_2-x_S/PEI). Copper sulfide acts as an antimicrobial agent through the direct toxic effects of its ions, its ability to enhance PTT and PDT via light absorption and its catalytic role in generating destructive hydroxyl radicals. The positive charges facilitated contact with negatively charged bacteria cell walls. The shell regulated the release of copper ions and ROS levels, Cu^2+^ led to bacterial enzymes and protein dysfunction and ROS enabled PDT, enhancing antibacterial activity. These combined effects, along with PTT, were responsible for the exceptional antibacterial properties of MPDA@Cu_2-x_S/PEI. They completely eradicated *E. coli* and nearly completely eradicated *S. aureus* [71]. In 2024, Yu et al. presented a dipolar-hollowed α-Fe_2_O_3_@Au/PDA nanospindle that consisted of iron oxide coated with Au and a PDA hybrid shell, which was then modified to obtain a dipolar-hollowed structure. This arrangement facilitated the loading of drugs, such as the photosensitizer zinc phthalocyanine and the antitumor drug doxorubicin, showing good drug loading–delivery behavior. This triple-mode therapy (PTT/PDT/Drug) offers an interesting therapeutic nanoplatform that can be applied for low-temperature antibacterial and drug delivery applications [72]. Instead, Liu et al. and Fang et al. utilized zinc oxide (ZnO) in conjunction with PDA in their antibacterial systems. The former expanded their antibacterial strategies by including quercetin, an antioxidant biomolecule, into their hydrogel. They developed a polyacrylamide–poly(2-acrylamido-2-methyl-1-propanesulfonic acid) hydrogel that included quercetin, quaternary ammonium salt chitosan and PDA-coated ZnO nanoparticles, and demonstrated a remarkable adhesion capability. The positively charged quaternary ammonium salt groups captured and destroyed the surface membrane of negatively charged bacteria. The hydrogel effectively disrupted bacterial enzyme activity by combining a mild photothermal effect (50 °C) with Zn^2+^ release. The quercetin component also provided stable free radical scavenging, ensuring the hydrogel’s antioxidant capacity [73]. Fang et al. developed an antibacterial phototherapeutic system that combined PDA, black phosphorus nanosheets (BP NSs) and ZnO nanowires (NW) on titanium (Ti) substrates. The combination of BP NSs and PDA enabled efficient photothermal therapy (PTT) under NIR irradiation. The photothermal effect helped to dissipate the biofilm and enhanced the Zn^2+^ release from ZnO, improving its antibacterial capability. The resulting platform demonstrated a synergistic “dissipating–killing” strategy, effectively dispersing bacterial biofilms and killing bacteria; this achieved an almost complete eradication of the biofilm in vivo, which is much better than that of PTT or ZnO NW alone [74].

Some research groups combined PTT with nitric oxide (NO) gas therapy to promote wound healing and antibacterial treatment. In 2021, Liu et al. developed a hybrid hydrogel consisting of two-dimensional polydopamine nanosheets loaded with BNN6, a NO donor embedded within a hydrogel that is obtained by crosslinking hydrazide-modified ***γ***-polyglutamic acid (***γ***-PGA-ADH) and aldehyde-terminated pluronic F127 (F127-CHO). The PDA nanosheets demonstrated excellent photothermal conversion, enabling bacterial killing through physical damage above 50 °C. Moreover, the generated heat triggered on-demand NO release. This hybrid hydrogel demonstrated an antibacterial effect both in vitro and in vivo. Notably, mouse infected wounds showed significant improvements when treated with hydrogels under NIR irradiation [75]. Two years later, the same approach was adopted by Zhang et al., who chose a chitosan/alginate hydrogel as a matrix, exploiting polysaccharides’ biomedical benefits. As previously described, PDA was used for photothermal conversion and BNN6 for NO release, but in this new study, bioactive glass (BG) nanoparticles were introduced, offering additional therapeutic benefits due to calcium and silicon ion release. PDA/BG NPs were also modified with β-cyclodextrin (β-CD) to improve the loading efficiency of BNN6 and provide an enhanced controlled release for NO [76]. The Gel/PDA@BNN6 hydrogel presented by Yang et al. in 2024, on the other hand, was based on methacrylate gelatin (GelMA), offering a unique three-dimensional porous structure and enhanced mechanical properties. This hydrogel was fabricated by incorporating PDA@BNN6 nanoparticles into GelMA through UV-induced polymerization. The resulting nanocomposite exhibited superior material stability, robust mechanical performance and favorable biocompatibility in vitro [77]. In the same year, Wang et al. integrated NO release and photothermal effects into a single nanoplatform for efficient antibacterial action. This nanoplatform comprised mesoporous polydopamine (MPDA) and sodium nitroferricyanide (III) dihydrate (SNP). The high NIR photothermal conversion ability of PDA induced SNP to generate NO. In this study, researchers employed a different NO releaser to kill bacteria and disrupt biofilms. The SNP/MPDA nanoplatform achieved a high bactericidal effect with a near-zero bacterial survival rate without the use of a hydrogel matrix [78]. Unlike the other hydrogels, which rely only on the therapeutic release of NO and photothermal effects, PHDNN6, developed by Jiang et al., integrated real-time diagnostic capabilities, allowing for the dynamic assessment of the wound microenvironment. To fabricate this new material, PDA@BNN6 NPs were embedded within a poly(N-isopropylacrylamide)-based hydrogel matrix with the addition of methacrylated and dopamine-grafted hyaluronic acid. The novel hydrogel combined wireless Bluetooth temperature monitoring with light-triggered nitric oxide (NO) release. In fact, changes in the rat wound temperature altered the temperature-responsive hydrogel volume, causing electrical resistance variations detectable by Bluetooth and transmitted wirelessly to a smartphone. In vivo and in vitro studies confirmed excellent biocompatibility, antibacterial properties, anti-inflammatory effects and accelerated wound healing [79].

As mentioned, the PHDNN6 hydrogel integrated real-time detection. In a similar approach, Jiang et al. developed a multifunctional theranostic nanoplatform that combined wound biofilm infection therapy with real-time detection. This nanoplatform was designed both for detecting wound infections and triggering ciprofloxacin (CIP) release for biofilm eradication via photothermal therapy. This system utilized mesoporous polydopamine nanoparticles grafted with rhodamine B. In the presence of a biofilm, lipase enzymes hydrolyzed the ester bond between the PDA and rhodamine B, restoring rhodamine fluorescence emissions. Subsequently, 808 nm irradiation efficiently initiated photothermal therapy and triggered CIP release, effectively eradicating the biofilm [80].

In a related approach, based again on CIP, Meng et al. developed a novel method to address the limitations of current antibacterial treatments by generating a microelectric field on nanoparticles. They designed Janus pyroelectric NPs that combined photothermal effects and a built-in pyroelectric field, which contributed to antibacterial activity through direct ablation and the electrodynamic production of ROS. These Janus NPs were created by partially coating PDA on tetragonal BaTiO_3_ (tBT) NPs and then loading CIP on the PDA caps. Upon NIR illumination, the PDA caps generated a photothermal effect that drove the NPs’ motion and created a pyroelectric effect in the tBT NPs. This design enhanced CIP release, disrupted bacterial membrane potentials and achieved synergistic antibacterial effects, demonstrating its potential for the effective treatment of bacterial infections [81].

In an alternative approach, Patil et al. combined the potential of PDA to unzip carbon nanotubes (uCNTs). CNTs have been largely used in biomedical applications due to their optimal mechanical properties and antibacterial activity. The uCNT-PDAs were then embedded within a polycaprolactone (PCL) nanofibrous membrane obtained by electrospinning. The nanofibers showed ROS-scavenging activity in the absence of NIR and the generation of free radicals in the presence of NIR light. PCL/uCNT@PDAs exposed to NIR laser irradiation showed good antibiofilm and antibacterial properties although less efficient compared to the other studies previously cited [82].

Alinezhad et al. focused mostly on the treatment of infected wounds by developing a photothermally active hydrogel containing platelet-rich plasma (PRP). The hydrogel was composed of alginate (Alg), gelatin (GT), PDA and PRP cross-linked with CaCl_2_. The resulting hydrogel showed improved strength, good swelling properties and high photothermal, antibacterial and antioxidant activities. The hydrogel also sustained the release of growth factors and demonstrated good hemocompatibility, cytocompatibility and hemostatic properties. In vitro tests revealed a decreased bacteria survival ratio when PDA was introduced. In vivo experiments demonstrated that the hydrogel effectively promoted infected wound healing by accelerating re-epithelialization, facilitating collagen deposition and enhancing angiogenesis [83].

Quni et al. faced the problem by means of a different approach. Indeed, they developed a nanoplatform by loading the flavonoid naringenin onto hollow mesoporous polydopamine NPs in a π−π-stacked configuration and subsequently encasing them within macrophage membranes. These membranes effectively neutralized inflammatory factors. The bacterial biofilm was then dismantled through the photothermal properties of PDA and the quorum-sensing inhibitory effects of naringenin [84]. Similarly, Jiang et al. employed a similar strategy, replacing naringenin with luteolin as the quorum-sensing inhibitor. Luteolin was loaded onto hollow mesoporous PDA NPs, which were subsequently coated with hyaluronic acid. Bacterial hyaluronidase enzymes degraded the outermost layer, and the acidic bacterial environment triggered luteolin release, effectively hindering and dispersing the biofilm. This release was accelerated by exposure to NIR radiation due to photothermal properties of polydopamine, which also aided in disrupting biofilm integrity by increasing temperature [85].

**Figure 7:**
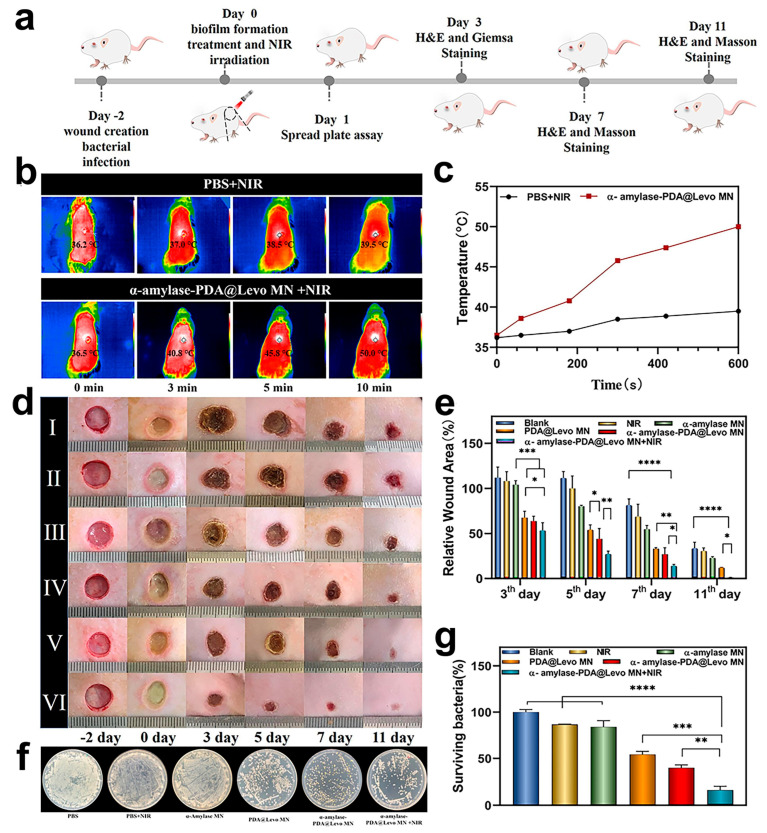
In vivo antibiofilm effect of different MNs. (**a**) The treatment scheme for antibiofilm activity assays in vivo. (**b**,**c**) Infrared thermography (1.0 W cm^2^, 10 min) and corresponding temperature curves of the rats during treatment. (**d**) Biofilm formation and wound photographs after different treatments for 3, 5, 7 and 11 days, respectively (groups I–VI represent Blank, NIR, α-amylase MN, PDA@Levo MN, α-amylase-PDA@Levo MN and α-amylase-PDA@Levo MN + NIR, respectively). (**e**) Relative wound area at different time points. (**f**,**g**) Representative photographs of culture plates showing surviving bacteria under various treatments. Statistical significance was set as *p* < 0.05 (“*”), *p* < 0.01 (“**”), *p* < 0.001 (“***”) and *p* < 0.0001 (“****”), respectively. Reprinted with permission from Ref. [86]. Copyright 2022 Elsevier.

Another approach was adopted by Yu et al., who designed a dissolvable microneedle patch loaded with α-amylase and levofloxacin-loaded PDA NPs (see Figure 7). The microneedles destroyed the physical barrier of biofilms and released the enzyme that degraded the extracellular polymeric substances (EPSs) of which the compact barrier was composed. Levofloxacin-loaded PDA NPs synergistically eradicated bacteria by combining antibiotics and mild photothermal therapy (PPT 50 °C) under 808 nm laser irradiation. Compared to other studies, in terms of bacterial eradication, these results are less impressive, but this still offers an extremely valid alternative. In fact, the treated wound was completely closed after 11 days, and the wound area was significantly smaller compared to that of the blank group. To sum up, this therapy shortened the period of inflammation and promoted wound healing and tissue regeneration [86].

Yang et al. identified a critical limitation in phototherapy-based antibacterial treatments: the bactericidal effect, dependent on continuous light irradiation, diminishes upon light removal, potentially leading to in situ bacterial repopulation. To assess this limitation, they proposed a self-rechargeable nanofibrous membrane. This system consisted of Mxene/Ag_3_PO_4_ bio-heterojunctions, functionalized with PDA and embedded within an electrospun poly(polycaprolactone) scaffold fibrous membrane. Under NIR illumination, the system showed powerful bactericidal effects through phototherapy and released Ag^+^ ions for metal ion therapy (MIT). Notably, PDA enabled the in situ reduction of Ag^+^ ions to Ag(0) nanoparticles when NIR light was absent, facilitating self-rechargeability. Subsequent NIR re-exposure triggered the ^1^O_2_-mediated oxidation of these Ag(0) nanoparticles, releasing Ag^+^ ions and sustaining the synergistic therapeutic effect. Consequently, the membrane demonstrated exceptional bactericidal efficacy and effectively eradicated biofilms through combined PTT, PDT and MIT [87].

A review of the recent scientific literature indicates that the applications of PDA extend beyond its antibacterial properties, with its use also demonstrating positive effects in diabetes therapy. In diabetic patients, a major issue is related to chronic unhealed wounds. Diabetes significantly impairs the ability of the body to heal wounds because of several factors. In particular, the high concentration of glucose in blood vessels damages them, hindering the transport of oxygen and essential nutrients to the wound site and worsening the wound healing process. Diabetic wounds are also highly inflamed and infected, factors that contribute to the difficulties mentioned above. The wound environment is usually acidic (pH around 4.5–6.5) and shows a higher temperature compared to nearby tissues. Therefore, several research projects have recently focused on developing multifunctional wound dressings for diabetic patients, combining glucose reduction activity, antibacterial activity, ROS scavenging activity and skin regeneration promotion. Hydrogel embedment perfectly matches the desired objectives since this matrix creates a moist environment for wounds, absorbs tissue exudates and can transport functional drugs to help wound healing.

In this regard, Hua and co-workers developed a multifunctional injectable hydrogel (QPTx) composed of phenylboronic-modified quaternized chitosan (QCS-PBA), polydopamine-coated tunicate cellulose nanocrystals (PDAn@TCNCs) and polyvinyl alcohol (PVA), exhibiting triple responsiveness to pH, temperature and glucose [88]. Zhu et al. introduced a multifunctional nanocomposite hydrogel (Met@CuPDA NPs/HG) fabricated using metformin-laden copper-loaded polydopamine nanoparticles (CuPDA NPs) embedded in a hydrogel matrix formed by gelatin modified with dopamine (Gel-DA) and hyaluronic acid modified with phenyl boronate acid (HA-PBA) [89]. Deng and colleagues developed a double-network hydrogel (FH–M@S) enhanced by SS31 (a mitochondria-targeted peptide)-loaded MPDA NPs and composed of pluronic F127 diacrylate (F127DA) and hyaluronic acid methacrylate (HAMA) [90]. These three studies positively highlight the photothermal antibacterial effects of hydrogels under NIR irradiation and the controlled release of the drug. In vitro and in vivo tests showed the hydrogels’ ability to scavenge ROS, enhance cell viability and migration and promote angiogenesis. Moreover, they accelerated wound closure, promoted tissue regeneration, enhanced vascularization and reduced inflammation compared to control groups. All the systems discussed in the above section are summarized in Table 2.

## 4. Other Applications

The approach of combining advanced biomaterials with photothermally controlled release mechanisms and multiple therapies has also been explored for the treatment of many different biological issues and diseases, in addition to those already cited.

For example, tissue repair and regeneration for the reconstruction of large-scale bone defects can largely benefit from the synergetic effects of on-demand drug delivery and mild heat stimulation. Bone defect repair, particularly in large-scale injuries, remains a major clinical challenge due to the limitations in current therapies, such as autologous and allogenous transplantation, which face issues like donor site morbidity, immune rejection and limited graft availability. Biomaterial-based scaffolds, while promising, often lack sufficient bioactivity and multifunctionality, factors that hinder their clinical translation. A critical barrier to effective bone regeneration is insufficient vascularization, which impairs nutrient and oxygen delivery, thereby limiting stem cell recruitment and osteogenesis. Combining biochemical and biophysical cues, NIR-responsive hydrogels with mild photothermal therapeutic activity are highly desirable for promoting bone regeneration by combining moderate hyperthermia and bioactive components [91].

Nevertheless, most hydrogel systems lack precise control over re-mineralization and suffer from poor mechanical properties, thereby hindering effective bone repair. This limits their ability to accurately reconstruct the osteovascularization network, crucial for successful bone regeneration. Wu et al. addressed this challenge by developing a novel NIR light-responsive intelligent hydrogel system (HG/MPa) to reconstruct the osteovascularization network through the spatiotemporal management of angiogenesis and osteogenesis. This system combines a hydroxypropyl chitosan/gelatin (HG) hydrogel with polydopamine-coated Ti_3_C_2_T_x_ MXene (MP) nanosheets loaded with an acidic fibroblast growth factor (aFGF). Upon NIR irradiation, the HG/MPa hydrogel achieves an initial fast release of aFGF to stimulate rapid angiogenesis. Simultaneously, the mild heat generated by the MXene nanosheets promotes osteogenic differentiation. In vitro and in vivo results showed HG/MPa’s thermal response allowed for controlled aFGF release and gentle heating, aligning with natural vascularization and bone formation. In particular, in vivo studies on rats with critical-sized bone defects revealed that the HG/MPa hydrogel, combined with mild photothermal treatment, significantly accelerated bone healing. This acceleration was achieved by increasing the density of the osteovascularization network, recruiting endogenous stem cells and facilitating the production of osteogenesis/angiogenesis-related factors [91]. In a similar approach, Ma et al. exploited photothermal therapy to enhance bone regeneration, but they also introduced a mussel-inspired multi-bioactive microsphere scaffold as a unique delivery system. This scaffold was composed of methacrylated silk fibroin (SFMA), magnesium ascorbyl phosphate (MAP) and PDA. The microspheres were fabricated using a microfluidic electrospray and coated with PDA for adhesion and photothermal conversion. MAP was encapsulated for sustained release, promoting the formation of a vascular network and osteogenic differentiation. The PDA coating enabled the microspheres to self-assemble into a scaffold in situ within bone defects and provided photothermal responsiveness for enhanced bone regeneration. In vitro experiments demonstrated that the scaffold promoted angiogenesis and bone marrow stromal cell proliferation and differentiation, while in vivo studies using a rat femoral defect model showed that bone defect healing was effectively enhanced. The synergistic effect of MAP release and mild photothermal stimulation significantly improved bone regeneration [92]. Moving beyond bone regeneration, Li et al. shifted their focus to the critical challenges of bacterial infection and poor osseointegration in titanium implants, introducing a novel approach that combined antibacterial drug delivery with photothermal therapy. A novel HAase/NIR-responsive surface was engineered on titanium by combining ciprofloxacin (CIP) with photothermal therapy. MPDA nanoparticles loaded with CIP (MPDA@CIP) were anchored onto the titanium surface and coated with sodium hyaluronate–catechol (HAc). The HAc layer inhibited initial bacterial adhesion but degraded in the presence of bacterial hyaluronidase, triggering CIP release at the infection site. NIR light irradiation enhanced both CIP release and the photothermal effect of MPDA. In vitro and in vivo assays demonstrated excellent antibacterial efficacy against *S*. *aureus* and MRSA. The modified titanium also exhibited promoted bone formation ability. The HAase-triggered and NIR-enhanced drug release mechanism guaranteed on-demand antimicrobial delivery. This functionalized titanium implant represents a promising approach to reduce post-operative infections and improve long-term implant success [93]. Expanding the application of these advanced material strategies beyond bone, Luo et al. demonstrated their potential in cartilage repair. These authors developed a multifunctional MPDA nanoparticle system, KGN@MPDA-PCM, for the near-infrared (NIR) photothermal controlled release of kartogenin (KGN) to enhance cartilage repair. Their system incorporates a phase-change material (PCM) as a gatekeeper for KGN release. Upon NIR irradiation, MPDA exhibited excellent photothermal conversion, melting the PCM and triggering the release of KGN, which promoted the chondrogenic differentiation and inhibited the hypertrophic differentiation of mesenchymal stem cells (MSCs). MPDA also exhibited good biocompatibility and antioxidant properties by scavenging ROS. In vivo experiments in a rat model with cartilage injuries showed that the intra-articular injection of KGN@MPDA-PCM combined with NIR irradiation significantly improved cartilage regeneration and subchondral bone repair [94]. In addition to tissue regeneration and infection control, other studies by Wang et al. and Chen et al. addressed the complex challenges of chronic joint diseases, but with a focus on distinct conditions: osteoarthritis (OA)and rheumatoid arthritis (RA), respectively. Wang et al. focused mostly on long-term, stable-release osteoarthritis drug delivery systems. These authors evaluated MPDA nanoparticles, known for their biocompatibility and high loading capacity, as a sustained drug carrier for osteoarthritis treatment. A drug-loaded microsphere system (RCGD423@MPDA) was developed and tested in vitro and in a rat model of osteoarthritis. RCGD423 is a small molecule that regulates osteoarthritis-induced inflammatory responses. The results showed that MPDA exhibited high drug retention, and the microsphere system provided controlled drug release for over 28 days. In vitro experiments demonstrated that this system inhibited apoptosis-related cartilage degeneration. In vivo, drug delivery via RCGD423@MPDA more effectively reduced cartilage damage and proteoglycan loss compared to a non-vectored drug. The developed system maintained a steady and controlled drug release, ensuring inflammatory regulation for a longer duration than traditional treatments. These findings suggest that MPDA can be an effective controlled-release carrier for inhibiting osteoarthritis progression [95]. Chen et al. addressed the challenges of treating RA, an autoimmune disease mainly caused by inflammatory cell infiltration (e.g., M1 macrophages), poor O_2_ supply at the joint and excess reactive oxygen species (ROS)-induced oxidative injury. The researchers developed a novel pH-responsive nanocomposite called CZP, composed of CeO_2_-doped zeolitic imidazolate framework-8 (ZIF-8) coated with PDA. Under NIR light irradiation, PDA acts as a photothermal agent to generate heat and as an ROS scavenger, increasing the temperature in the inflamed area and reducing oxidative stress. This temperature rise also promotes the breakdown of the acidic-responsive ZIF-8, leading to the release of CeO_2_ nanoparticles, which then catalyze the production of oxygen to alleviate hypoxia. In vitro experiments demonstrated that CZP was effectively taken up by macrophages, exhibited ROS scavenging and oxygen-generating activities and induced the photothermal killing of inflammatory cells. In vivo studies using adjuvant-induced arthritis (AIA) rat models showed that CZP combined with NIR laser irradiation significantly suppressed inflammation, reduced joint damage and lowered the expression of pro-inflammatory cytokines and HIF-1α [96].

Che et al. focused on applications for osteoporosis, a bone disease characterized by bone loss and microarchitectural deterioration. This study presents a biomimetic and bioactive scaffold designed for the intelligent pulsatile delivery of teriparatide, a drug used to treat both local osteoporotic bone defects and systemic osteoporosis. The platform consisted of a mesoporous bioglass (MBG) scaffold coated with PDA and loaded with thermo-sensitive liposomes (TSLs) containing teriparatide, devised to achieve a non-invasive and controllable drug delivery system. PDA acts as a heater triggered by NIR irradiation, inducing a phase transition in TSLs and a pulsatile release of teriparatide. In vitro experiments demonstrated controllable pulsatile release for 14 days and an enhanced osteogenic differentiation of bone marrow stromal cells. In vivo studies proved the local bone regeneration of a cranial defect model in osteoporotic rats, and an analysis of the femur and lumbar vertebra showed systemic anti-osteoporosis effects. The system allowed for on-demand drug release, overcoming the limitations of traditional delivery methods [97].

The last reported studies were mostly focused on bone- and joint-related issues and diseases, but the same approaches have been extended to other injuries.

**Figure 8:**
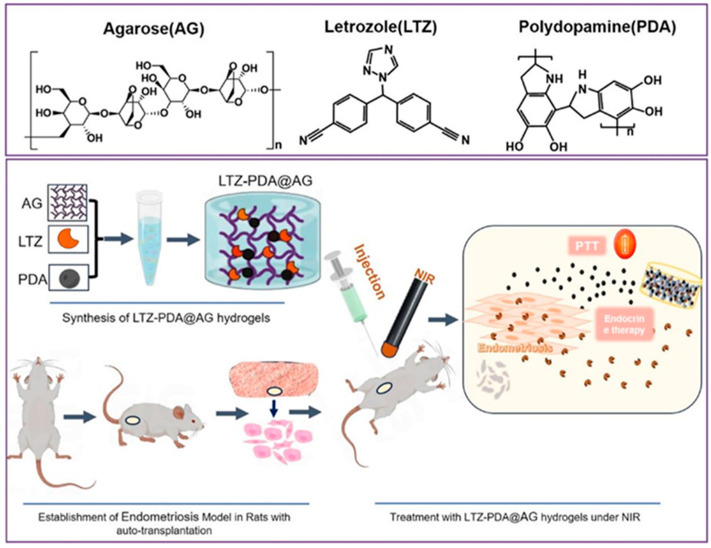
Synthesis of LTZ-PDA@AG hydrogels and endocrine therapy on endometriosis under NIR. Reprinted from Ref. [98].

One of them is endometriosis, a chronic and often painful condition where tissue similar to the lining of the uterus (the endometrium) grows outside of the uterus. This misplaced tissue can be found on the ovaries, fallopian tubes and outer surface of the uterus. It is normally treated with endocrine therapies, which are based on aromatase inhibitors, and drugs that reduce estrogen production in the body. However, these drugs display poor therapeutic effects with a high recurrence probability. Thus, novel strategies for the treatment of endometriosis are being investigated. The recent research of Tian et al. introduced a novel injectable hydrogel (LTZ-PDA@AG) for endometriosis treatment, combining photothermal and endocrine therapies. The hydrogel is composed of PDA, letrozole (LTZ) and agarose (AG). The release of letrozole, an aromatase inhibitor, can be precisely controlled by NIR light, allowing for targeted drug delivery. PDA allows efficient photothermal therapy to destroy endometriotic cells (see Figure 8). In vitro tests showed the hydrogel’s ability to kill endometrial stromal cells (ESCs) under NIR irradiation. In vivo experiments on a rat model of endometriosis demonstrated that the LTZ-PDA@AG hydrogel significantly reduced the volume of endometriotic foci when it was combined with NIR irradiation, highlighting the potential of this NIR-responsive hydrogel for clinical translation in endometriosis therapy [98].

Epilepsy is a chronic brain disorder characterized by recurrent and unprovoked seizures, sudden surges of electrical activity in the brain that can cause a variety of symptoms, including convulsions, temporary confusion or loss of awareness. Commonly, antiepileptic drugs are employed to treat this disease, even if they suffer from insufficient brain targeting induced by the blood–brain barrier and several adverse effects. To address these limitations, Wu and colleagues designed a nanoengineered drug delivery system (PPY-PDA-PHT-ANG) combining polypyrrole (PPY) and PDA nanoparticles loaded with phenytoin (PHT) and functionalized with an angiopep-2 (ANG) peptide for synergistic brain targeting. The nanoparticles exhibited electroresponsive drug release triggered by epileptiform discharges and enhanced blood–brain barrier (BBB) penetration facilitated by ANG modification and the NIR light-induced photothermal conversion of PDA. In vitro studies confirmed the improved conductivity and sustained drug release of the nanoparticles under electrical stimulation, as well as enhanced BBB penetration with ANG modification and NIR irradiation. In vivo experiments demonstrated that the delivery system effectively inhibited seizures at a lower drug dosage compared to conventional administration [99].

Venous thrombosis is a condition where a blood clot (thrombus) forms in a vein, blocking the flow of blood. To date, thrombolytic drugs are the only treatments applied for this disease, but they can have numerous adverse effects involving hemorrhagic complications and neurotoxic effects. Thus, a new strategy to achieve effective and safe thrombosis therapy is required. Li et al. developed a biomimetic nanoplatform (HMPC@PM) engineered with a platelet membrane to target and treat venous thrombosis. The nanoplatform was composed of a melanin core and a porphyrin-based covalent organic framework (Por-COF) shell loaded with hirudin. Under NIR light irradiation, the nanoplatform generated heat and ROS, enabling PTT/PDT for effective thrombolysis. In vitro experiments demonstrated efficient blood clot lysis and inhibition of platelet aggregation. In vivo studies on a mouse model of venous thrombosis showed that HMPC@PM effectively targeted and dissolved thrombi under NIR irradiation. The loaded hirudin provided long-term anticoagulation, preventing thrombus recurrence without causing thrombocytopenia [100]. All the systems discussed in the above section are summarized in Table 3.

## 5. Future Perspectives

The examples reported in this review paper demonstrate how the most promising therapeutic and theranostic nanoplatforms are designed to combine multiple approaches in a synergistic way. In this framework, the multifunctionality of melanin, combined with its intrinsic biocompatibility, is a very promising tool for its application in nanomedicine. However, the existing studies still present some general drawbacks, which we chose to summarize in this section rather than repeating them when analyzing each case. In particular, reviewing the most recent examples of melanin-based nano-systems that show an enhanced release of therapeutics upon light irradiation, we noticed that the actual mechanism of the release is usually not analyzed in detail. The research is very focused on the design and application of quite complex platforms that combine multiple therapies and demonstrate their actual effectiveness. From an applicative point of view, this is surely an efficient approach; nevertheless, the complexity of the proposed systems makes it often difficult to understand how they really work. What happens to these nano-systems when they are irradiated? Why does light irradiation enhance the release of therapeutics? Studies and calculations performed for metallic plasmonic nanostructures demonstrate that the increase in local temperature is almost negligible in the case of poorly focused and non-pulsed light irradiation [101,102,103]. Similar results are expected for melanin-based nanostructures. Hence, we believe that future research should consider in more detail the investigation of the mechanism behind photothermal release considering the following points.

### 5.1. What Local Temperature Is Reached?

The irradiation of the melanin-based nanoplatform is expected to cause an increase in the local temperature because of the PT effect but the actual temperature increase strongly depends on important experimental parameters such as local irradiance, which depends on the kind of focus and can change considerably in time, especially when pulsed sources are utilized. Local changes in temperature need to be accurately considered, especially because they are driven by the kinetics of heating up and of heat dissipation into the surrounding medium. As a consequence, the performances of different therapeutic nanoplatforms are strongly affected by the experimental setup used for their photoactivation. Detecting the actual local temperature increase in the operative conditions is thus very important. On the other hand, we are aware that methods for determining the local temperature with at least micrometric resolution are not simple to apply. One relatively simple approach is based on the optical detection of the fluorescence of a molecular thermometer in the proximity of the photoactivable nano-systems [104,105]. An advantage of this approach is the possibility to use the same light source for activating the photothermal release and exciting the fluorescent molecules, but this requires NIR absorption and quite photostable molecules for temperature detection. Possible fluorescence quenching should also be considered. Other techniques for measuring local temperature are based on the detection of changes in the properties of the surrounding medium, such as refractive index, but also, in this case, their application is far from being trivial. In general, we believe that new, simple methodologies for detecting temperature in the micrometric to nanometric scale need to be developed, and that these will give a great contribution to our understanding of photothermal release processes [106].

### 5.2. How Does Local Temperature Change over Time?

Although the mechanism of photothermal release in many cases has not been investigated in detail, it is well known that in general, any chemical process, including molecular release, is accelerated by an increase in temperature. On the other hand, the dependency of the rate of a chemical reaction on the temperature is not linear but, according to the Arrhenius equation, exponential. This becomes extremely important when the temperature fluctuates over time. Knowing the average temperature across time gives poorly useful information for investigating photothermal release. Hence, the local temperature should be detected not only with a high spatial resolution but also with a good temporal resolution. Temperature fluctuations are expected to become particularly relevant in the case of pulsed excitation.

### 5.3. Are Different Experimental Results Comparable?

While the comparison of different experiments in the same framework (same publication or set of publications by the same laboratory) is possible, comparing experiments performed in different conditions can be complicated. This is because, as discussed above, performances strongly depend on the experimental setup. For this reason, assessing which nanoplatform behaves better, as far as photothermal release is concerned, is not trivial. We believe that, in general, a standardized setup should be identified to compare the different nano-systems. Of course, we are aware that optimal performance also depends on the optimization of the setup, but testing the different nano-systems in standardized conditions may also help to design a new setup to optimize their performance.

### 5.4. The Role of Chemistry and Photochemistry

While local chemical parameters such as the local acidic pH in cancer tissue have been exploited as triggers to activate or accelerate the photothermal release by melanin-based nanostructures, the effect of irradiation on the local chemical properties typically is not investigated in detail. Different kinds of melanin are known to be photoreactive, even if their photoreactivity is poorly investigated. Some components of melanin may also behave as a photo-acid or photo-base, or as a photo-acid generator (PAG) or photo-base generator (PBG). In general, these processes are expected to enhance or inhibit molecular release. This possible effect should be considered in combination with photothermal release.

## 6. Conclusions

In this review paper, we analyzed and critically discussed the most recent examples of nano-systems based on melanin-related NPs used for photothermal release in biological applications. Melanin, and particularly its biomimetic analogue polydopamine, is finding increasing applications in nanomedicine, especially due to its high biocompatibility, versatility and optical properties. The irradiation of these materials with NIR light is known to produce an effective photothermal effect, which is exploited by several authors to enhance the release of therapeutic agents in order to combine PTT with CT. As discussed in Section 5, we believe the complete understanding of the actual behavior of the examined nano-systems requires, in many cases, more detailed and basic investigations. Nevertheless, in general, we can conclude that the integration of melanin-like materials is very promising for the design of innovative, highly biocompatible tools for nanomedicine.

## Figures and Tables

**Table 1:** Summary of the systems discussed in Section 2.

Disease	Imaging	Agents	Reference
Melanoma	-	Met and Ammonia Borane	[32]
Non-small cell lung cancer	FL	DOX	[33]
Osteosarcoma	-	DOX	[34]
Colon cancer	FL	DOX	[35]
Melanoma	-	DOX	[36]
Cancer	-	DOX	[37]
Skin tumors	-	Melanin NP, SiO_4_^4−^	[38]
Breast cancer	Fluorescence	DOX	[39]
Breast cancer	-	ACGs	[40]
Lung cancer	-	BA	[42]
Breast cancer	-	Curcumin	[43]
Breast cancer	Photothermal	DOX	[44]
Anaplastic thyroid carcinoma	FL/Photothermal	DOX, double-stranded CpG oligodeoxynucleotides	[45]
Cancer	-	T7 polypeptide-engineered bacterial outer membrane vesicles	[47]
Osteosarcoma	-	L-Arg, ICG, H_2_O_2_	[48]
Breast cancer	-	Ce6, PTX	[49]
Breast cancer	Photothermal	ZnPc, SPC	[50]
Breast cancer	FL	CsPbBr3 quantum dots	[51]
Breast cancer	FL, Photothermal	CuO_2_	[52]
Breast cancer	-	DOX	[53]
Cancer	MR	DOX	[54]
Cancer	MR	Manganese sulfide nanoclusters, H2S	[55]
Breast cancer	MR	DOX	[56]
Breast cancer	FL/MR	MnCO_3_-mineralized PDA NP	[57]
Breast cancer	FL	DOX	[58]
Colon carcinoma	Ultrasound/PA	GOx, PFP	[59]
Breast cancer	Photoacoustic	DOX	[60]
Triple-Negative Breast Cancer	Ultrasound	CQ, ICG	[61]

**Table 2:** Summary of the systems discussed in Section 3.

Matrix	Agents	Reference
Hydrogel	AgNPs	[62]
Hydrogel	AgNPs	[63]
Hydrogel	rGO/BG/AgNC	[64]
-	MOFs/AgNPs	[65]
-	MOFs/AgNPs	[66]
Hydrogel	Ag Nanomotors	[67]
Hydrogel	AgNPs/Levofloxacin	[68]
Hydrogel	AgNPs/Lidocaine	[69]
Hydrogel	Ketoprofene	[70]
-	Cu_2-x_S NP	[71]
-	α-Fe_2_O_3_@Au NPs	[72]
Hydrogel	ZnO NPs, quercetin, quaternary ammonium salt chitosan	[73]
Ti substrates	BP NS, ZnO NW	[74]
Hydrogel	BNN6 (NO donor)	[75]
Hydrogel	BNN6 (NO donor)/BG NPs	[76]
Hydrogel	BNN6 (NO donor)	[77]
Hydrogel	SNP (NO donor)	[78]
Hydrogel	BNN6 (NO donor)	[79]
-	Ciprofloxacin	[80]
-	Janus pyroelectric NPs, ciprofloxacin	[81]
Electrospun polycaprolactone	uCNT	[82]
Hydrogel	PRP	[83]
Macrophage membrane	Naringenin	[84]
Hyaluronic acid coating	Luteolin	[85]
Microneedle patches	Levofloxacin, α-amylase	[86]
Electrospun polycaprolactone	Mxene/Ag_3_PO_4_	[87]
Hydrogel	QCS-PBA, tunicate cellulose crystals, insulin drugs	[88]
Hydrogel	Metformin	[89]
Hydrogel	SS31	[90]

**Table 3:** Summary of the systems discussed in Section 4.

Disease	Agents	Reference
Bone regeneration	MXene NS, aFGF	[91]
Bone regeneration	MAP	[92]
Bacterial infection and poor osseointegration in titanium implants	Ciprofloxacin	[93]
Cartilage repair	Kartogenin	[94]
Chronic joint osteoarthritis	RCGD423	[95]
Chronic joint rheumatoid arthritis	CeO_2_-doped ZIF-8	[96]
Osteoporosis	Teriparatide	[97]
Endometriosis	LTZ	[98]
Epilepsy	PHT	[99]
Venous thrombosis	Hirudin	[100]

## Data Availability

No new data were created or analyzed in this study. Data sharing is not applicable to this article.
